# The Impact of Mismatch Repair Status on Prognosis of Patients With Gastric Cancer: A Multicenter Analysis

**DOI:** 10.3389/fonc.2021.712760

**Published:** 2021-11-25

**Authors:** Wen-Long Guan, Yue Ma, Yue-Hong Cui, Tian-Shu Liu, Yan-Qiao Zhang, Zhi-Wei Zhou, Jian-Ying Xu, Li-Qiong Yang, Jia-Yu Li, Yu-Ting Sun, Rui-Hua Xu, Feng-Hua Wang, Miao-Zhen Qiu

**Affiliations:** ^1^ Department of Medical Oncology, Sun Yat-Sen University Cancer Center, State Key Laboratory of Oncology in South China, Collaborative Innovation Center for Cancer Medicine, Guangzhou, China; ^2^ Department of Gastrointestinal Medical Oncology, Harbin Medical University Cancer Hospital, Harbin, China; ^3^ Department of Medical Oncology, Zhongshan Hospital, Fudan University, Shanghai, China; ^4^ Department of Gastric Surgery, Sun Yat-Sen University Cancer Center, State Key Laboratory of Oncology in South China, Collaborative Innovation Center for Cancer Medicine, Guangzhou, China

**Keywords:** MMR, MSI, gastric cancer, adjuvant chemotherapy, prognosis

## Abstract

**Background:**

The clinical role of deficient DNA mismatch repair (dMMR)/microsatellite instability-high (MSI-H) in gastric cancer (GC) is still controversial. We aimed to analyze the relationship between dMMR/MSI-H and clinicopathological features along with survival.

**Methods:**

Patients who were diagnosed with GC at the three big cancer centers in China from 2015 to 2020 were evaluated retrospectively. MMR/MSI status was assessed using immunohistochemistry/PCR. Clinical and pathological data were collected from the medical record system.

**Results:**

A total of 196 patients with dMMR/MSI-H status were enrolled for analysis. The prevalence of MSI-H/dMMR in GC was 6.6%. Another 694 proficient MMR (pMMR) GC patients were enrolled for comparison. Compared with pMMR patients, dMMR/MSI-H patients were associated with older age, female predominance, distal location in the stomach, earlier TNM stage, intestinal subtype, better differentiation, and more negative HER2 status. The median overall survival (OS) of the dMMR/MSI-H group was better than that of the pMMR/microsatellite stability (MSS) group (not reached *vs*. 53.9 months, *p* = 0.014). Adjuvant chemotherapy had no impact in both disease-free survival (DFS) and OS of dMMR/MSI-H patients (*p* = 0.135 and 0.818, respectively). dMMR/MSI-H patients had poorer response and progression-free survival (PFS) of first-line chemotherapy, though they were statistically significant (*p* = 0.361 and 0.124, respectively).

**Conclusions:**

dMMR/MSI-H GC patients have specific clinicopathological characteristics and better prognosis than pMMR patients.

## Introduction

Gastric cancer (GC) is one of the most common causes of cancer-related mortality worldwide. As a heterogeneous disease, patients with the same TNM stage and histological characteristics may respond differently to treatment and have different survival. Hence, specific classification was presented for guidance of clinical decision making. The Cancer Genome Atlas (TCGA) Research Network has identified four distinct molecular subtypes of GC through molecular evaluation of 295 GC patients: Epstein-Barr virus (EBV) positive, microsatellite instability (MSI), chromosomal instability (CIN), and genomically stable (GS) (1). As a result of dysfunction of mismatch repair (MMR), MSI leads to increased rate of replication error and hypermutational status, which results in increased probability of mutations in oncogenes or tumor suppressors. MSI status is commonly assessed using PCR. MMR proteins, including FmutL homologue 1 (MLH1), mutS homologue 2 (MSH2), mutS homologue 6 (MSH6), and PMS1 homologue 2 (PMS2), were determined by immunohistochemical (IHC) analysis. The concordance between MSI-high (MSI-H) status and deficiency of MMR protein function (dMMR) was 97.6%–99% (2, 3).

According to the data of TCGA, 21.7% patients of GC were identified as MSI. However, it did not distinguish between MSI-H and MSI-low (MSI-L). The prevalence of MSI-H/dMMR status in GC ranged from 8% to 25% in previous reports (4–14). MSI-H/dMMR status was a predictive marker of response to immunotherapy (15). Besides, MSI-H/dMMR status has been reported to be a prognostic and predictive factor in the adjuvant setting. In colorectal cancer, MSI-H/dMMR tumor shows better prognosis, and may be associated with lack of benefit from adjuvant chemotherapy in stage II disease (16, 17). Similar results were reported in GC patients (14). Recently, an individual patient data meta-analysis from four large randomized clinical trials performed in patients with resectable GC (MAGIC, CLASSIC, ARTIST, and ITACA-S) showed that patients with MSI-H/dMMR GC did not benefit from adjuvant chemotherapy after radical surgery (14). However, in this pooled analysis of four clinical trials, the number of MSI GC patients was still relatively low (N = 121), which made the statistical power limited. Moreover, due to the low prevalence of MSI-H/dMMR in GC, the clinical and pathological features, response to chemotherapy, and overall survival (OS) are still controversial.

With this in mind, we conducted this retrospective study, enrolling MSI-H/dMMR GC patients from three big cancer centers in China. By this, we tried to expand the sample size and explore the clinicopathological characteristics and predictive and prognostic values of MSI-H/dMMR status for GC.

## Methods

### Patients

Patients who were diagnosed as GC at the three big cancer centers in China (Harbin Medical University Cancer Hospital, Fudan University Zhongshan Hospital, and Sun Yat-sen University Cancer Center) from 2015 to 2020 were evaluated retrospectively. These three hospitals all have large patient volume in China. The studies involving human participants were reviewed and approved by the ethics committee of each hospital. All the patients were diagnosed as GC by H&E staining and histological analysis. The stage was determined using the American Joint Committee on Cancer (AJCC) TNM 8th stage system. Clinical data, including sex, age, family history, and primary tumor location, were collected from the medical record system. Patients with dMMR/MSI-H status were enrolled for analysis (a total of 196 cases, including 72 from Harbin, 71 from Shanghai, and 53 from Guangzhou). Besides, 694 cases with proficient MMR (pMMR) status diagnosed at Sun Yat-sen University Cancer Center in the same period were enrolled as comparison.

### Mismatch Repair/Microsatellite Instability Assessment

For MMR protein IHC analysis, 4-μm formalin-fixed, paraffin-embedded sections were prepared from the tissue blocks and stained for the MLH1, MSH2, MSH6, and PMS2 proteins. Primary antibodies included anti-MLH1 (M1, Ventana, USA), anti-MSH-2 (RED2, ZSGB-bio, China), anti-MSH6 (SP93, Ventana, USA), and anti-PMS2 (EP51, Dako, Denmark). Loss of MMR protein expression was designated when none of the neoplastic epithelial cells had nuclear staining, while positive internal control nuclei (lymphocytes and stromal cells) were present in the immediate vicinity of the tumor infiltrate. Normal expression was defined as the presence of nuclear staining of tumor cells irrespective of the proportion or intensity. A case was classified as dMMR if tumor cell nuclei were negative for one or the four MMR proteins in the presence of positively stained lymphocytes or fibroblasts as internal control. pMMR was defined if tumor cell nuclei, irrespective of the number or intensity, were positive for all MMR proteins tested.

The MSI status was evaluated using PCR. DNA was extracted from paired normal/tumor tissues that were formalin-fixed, paraffin embedded. Then PCR amplification was performed for two mononucleotide repeat markers (BAT25 and BAT26) and three dinucleotide markers (D5S346, D2S123, and D17S250) (11). MSI-H was defined as two or more markers mentioned above with instability. Otherwise, it was defined as microsatellite stability (MSS).

### Statistical Analysis

The patients’ clinicopathological features were summarized with descriptive statistics. Categorical variables were compared using chi-square test, and comparisons of continuous variables were performed using Student’s t-test. Five-year cause-specific survival (CSS) was calculated from the date of diagnosis to the date of cancer-specific death. Survival among different variables was compared using the Kaplan–Meier estimates and the log-rank test. Statistical analysis was carried out by the IBM SPSS Statistics 22.0.0 package software (SPSS Inc.) and the Intercooled Stata 13.0 (Stata Corporation, College Station, TX). All the *p*-values were two-sided, and statistical significance was set at *p* < 0.05.

## Results

### Clinical and Pathological Features

This study was composed of 196 cases of MSI-H/dMMR GC (72 from Harbin, 71 from Shanghai, and 53 from Guangzhou). The prevalence of MSI-H/dMMR in GC was 6.6% in total (8.3% in Harbin, 4.7% in Shanghai, and 6.8% in Guangzhou). The median age was 64 years (ranged from 31 to 87 years). There were 108 (55.1%) male and 88 (44.9%) females. The number of patients from TNM stage I to IV was 40 (20.4%), 61 (31.1%), 58 (29.6%), and 34 (17.3%), respectively. Most of their GC was located at the distal stomach (131, 66.8%). For Lauren classification, there were 40 (20.4%) cases with diffuse type, 73 (37.2%) with intestinal type, and 51 (26.0%) with mixed type. Five (2.6%) patients were HER2 positive, and eight (4.1%) cases were EBER positive. The clinical and pathological characteristics of MSI-H/dMMR GC are shown in **Table 1**. Compared with pMMR patients, dMMR/MSI-H patients were associated with some specific clinical and pathological features, including older age, higher proportion of female, distal location in the stomach, earlier TNM stage, intestinal histotype, better differentiation, and more negative HER2 status (**Table 1**, all *p* < 0.05).

**Table 1:** Clinical and pathological characteristics of dMMR/MSI-H gastric cancer.

Variables	N (%)		*p*
	dMMR	pMMR	
Site			
Harbin	72 (36.7)		
Shanghai	71 (36.2)		
Guangzhou	53 (27.0)	694 (100)	
Gender			**0.003**
Male	108 (55.1)	462 (66.6)	
Female	88 (44.9)	232 (33.4)	
Age	63.8 ± 10.5	57.0 ± 12.5	**<0.001**
Family history			**<0.001**
Yes	18 (9.2)	144 (20.7)	
No	174 (88.8)	522 (75.2)	
NA	4 (2.0)	28 (4.0)	
Tumor location			**<0.001**
Proximal or EGJ	12 (6.1)	229 (33.0)	
Middle	44 (22.4)	157 (22.6)	
Distal	131 (66.8)	267 (38.5)	
Whole stomach	5 (2.6)	6 (0.9)	
Others	2 (1.0)	17 (2.4)	
NA	2 (1.0)	18 (2.6)	
T stage			**<0.001**
T1	29 (14.8)	82 (11.8)	
T2	28 (14.3)	50 (7.2)	
T3	83 (42.3)	209 (30.1)	
T4	33 (16.8)	222 (32.0)	
Tx	23 (11.3)	131 (18.9)	
N stage			**<0.001**
N0	66 (33.7)	139 (20.0)	
N1	43 (21.9)	94 (13.5)	
N2	32 (16.3)	112 (16.1)	
N3	29 (14.8)	207 (29.8)	
Nx	26 (13.3)	142 (20.5)	
M stage			**<0.001**
M0	164 (83.7)	454 (65.4)	
M1	32 (16.3)	240 (34.6)	
TNM stage			**<0.001**
I	40 (20.4)	89 (12.8)	
II	61 (31.1)	130 (18.7)	
III	58 (29.6)	235 (33.9)	
IV	34 (17.3)	240 (34.6)	
NA	3 (1.5)	0 (0)	
Pathology			**<0.001**
Adenocarcinoma	168 (85.7)	530 (76.4)	
Signet ring	2 (1.0)	23 (3.3)	
Mix (adeno+signet)	11 (5.6)	121 (17.4)	
Others	12 (6.1)	20 (2.9)	
NA	3 (1.5)	0 (0)	
Differentiation			**0.005**
High/moderate	112 (57.1)	331 (47.7)	
Low/undifferentiated	69 (35.2)	331 (47.7)	
NA	15 (7.7)	32 (4.6)	
Lauren classification			**0.017**
Diffuse	40 (20.4)	224 (32.3)	
Intestinal	73 (37.2)	222 (32.0)	
Mix	51 (26.0)	171 (24.6)	
NA	32 (16.3)	77 (11.1)	
HER2 status			**0.001**
Negative	159 (81.1)	561 (80.8)	
Positive	5 (2.6)	76 (11.0)	
NA	32 (16.3)	57 (8.2)	
EBERs			0.802
Negative	100 (51.0)	508 (73.2)	
Positive	8 (4.1)	36 (5.2)	
NA	88 (44.9)	150 (21.6)	

dMMR, deficient DNA mismatch repair; MSI-H, microsatellite instability-high; pMMR, proficient mismatch repair; EGJ, esophagogastric junction. Bold value means that the p value was <0.05.

### Mismatch Repair Expression Mode

The detail of MMR protein expression mode is shown in **Table 2**. Most common defective expression was seen in MLH1 and PMS2 (153, 78.1%). Twenty-two cases (11.2%) only negatively expressed PMS2, and eight cases (4.1%) had concurrent loss of MSH2 and MSH6. We also found two cases that were pMMR but turned out to be MSI-H using PCR.

**Table 2:** The detail of expression pattern of 196 dMMR/MSI-H cases.

Markers	MLH1	MSH2	MSH6	PMS2	N (%)
Expression	(−)	(+)	(+)	(−)	153 (78.1)
	(+)	(+)	(+)	(−)	22 (11.2)
	(+)	(−)	(−)	(+)	8 (4.1)
	(+)	(−)	(+)	(−)	2 (1.0)
	(−)	(+)	(+)	(+)	2 (1.0)
	(−)	(−)	(−)	(+)	1 (0.5)
	(−)	(−)	(+)	(−)	1 (0.5)
	(+)	(+)	(+)	(+)	2 (1.0)*
	NA	NA	NA	NA	5 (2.6)*

dMMR, deficient DNA mismatch repair; MSI-H, microsatellite instability-high.

*The two cases with pMMR and five cases with unknown MMR status were confirmed as MSI-H using PCR.

### Survival of Patients With Deficient Mismatch Repair/Microsatellite Instability-High Gastric Cancer

**Figure 1:**
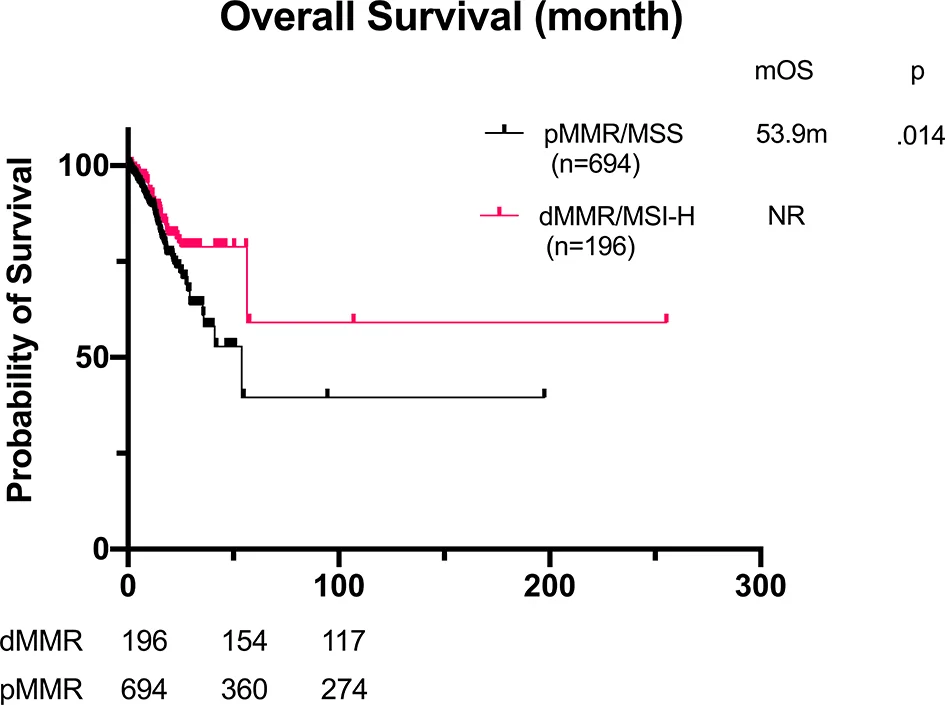
Overall survival of GC patients with dMMR/MSI-H (n = 196) and pMMR (n = 694). mOS: not reached *vs*. 53.9 months, *p* = 0.014. GC, gastric cancer; dMMR, deficient DNA mismatch repair; MSI-H, microsatellite instability-high; pMMR, proficient mismatch repair; mOS, median overall survival.

**Figure 2:**
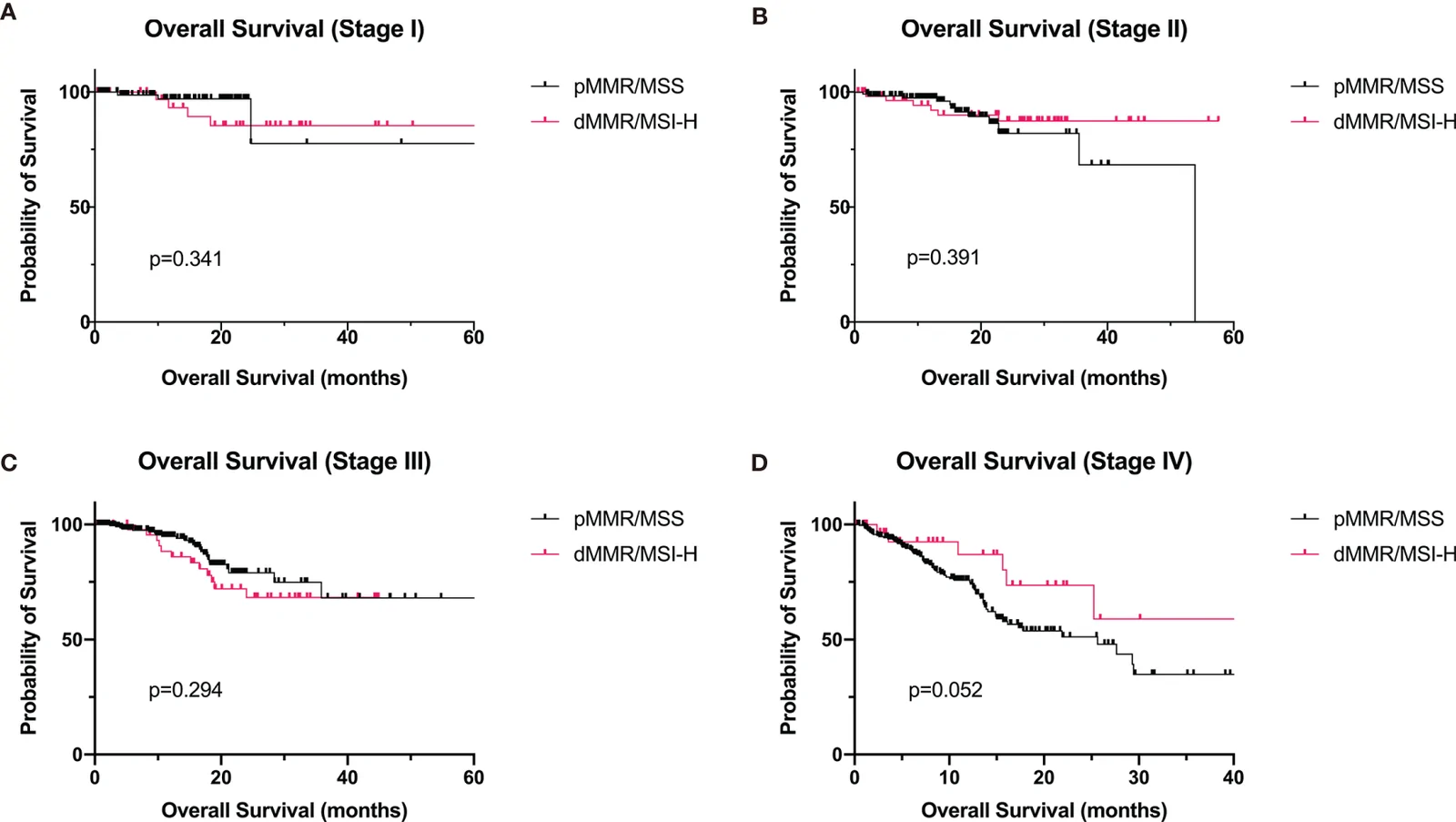
Overall survival of GC patients with dMMR/MSI-H and pMMR at stages I–IV. **(A)** Stage I (dMMR/MSI-H: n = 40; pMMR: n = 89). **(B)** Stage II (dMMR/MSI-H: n = 59; pMMR: n = 130). **(C)** Stage III (dMMR/MSI-H: n = 51; pMMR: n = 235). **(D)** Stage IV (dMMR/MSI-H: n = 32; pMMR: n = 240). GC, gastric cancer; dMMR, deficient DNA mismatch repair; MSI-H, microsatellite instability-high; pMMR, proficient mismatch repair.

The median OS of dMMR/MSI-H group was significantly better than that of the pMMR/MSS group (not reached *vs*. 53.9 months, *p* = 0.014, **Figure 1**). We examined the outcomes stratified by TNM stages. We found that the OS was not remarkably different in each TNM stage group (**Figure 2**). In stage IV, the OS of dMMR/MSI-H patients was numerically better than that of pMMR/MSS patients (56.5 *vs*. 25.6 months, *p* = 0.052), but it was not statistically significant. The multivariate analysis showed that TNM stage was the only prognostic factor associated with OS (**Table 3**).

**Table 3:** Univariable and multivariable Cox proportional hazard models for overall survival.

Variables	Univariate analysis		*p*	Multivariate analysis		*p*
	HR	95% CI		HR	95% CI	
Age			0.383			
<60	1					
≥60	0.863	(0.620, 1.201)				
Gender			0.078			
Male	1					
Female	1.352	(0.966, 1.891)				
Family history			0.110			
No	1					
Yes	1.401	(0.927, 2.118)				
Location						
Proximal or EGJ	1					
Middle	1.323	(0.834, 2.099)	0.234			
Distal	0.790	(0.515, 1.211)	0.279			
Whole stomach	1.452	(0.445, 4.739)	0.537			
Others	0.247	(0.034, 1.815)	0.170			
TNM stage						
I	1			1		
II	1.642	(0.686, 3.934)	0.266	2.380	(0.791, 7.162)	0.123
III	2.720	(1.212, 6.105)	**0.015**	3.597	(1.262, 10.252)	**0.017**
IV	8.976	(4.133, 19.495)	**<0.001**	10.864	(3.896, 30.294)	**<0.001**
Pathology						
Adenocarcinoma	1					
Signet ring	2.147	(0.996, 4.628)	0.051			
Mix (adeno+signet)	1.358	(0.862, 2.137)	0.187			
Others	1.873	(0.911, 3.850)	0.088			
Differentiation			**<0.001**			0.305
High/moderate	1			1		
Low/undifferentiated	2.077	(1.459, 2.957)		1.325	(0.774, 2.269)	
Lauren classification						
Diffuse	1			1		
Intestinal	0.506	(0.334, 0.767)	**0.001**	0.842	(0.464, 1.528)	0.571
Mix	0.525	(0.327, 0.841)	**0.007**	0.750	(0.452, 1.243)	0.264
MMR/MS status			**0.015**			0.930
dMMR/MSI-H	1			1		
pMMR/MSS	1.678	(1.104, 2.550)		0.979	(0.607, 1.579)	
HER2 status			0.404			
Negative	1					
Positive	1.259	(0.734, 2.159)				
EBERs			0.284			
Negative	1					
Positive	0.575	(0.210, 1.580)				

HR, hazard ratio; EGJ, esophagogastric junction; MMR, mismatch repair; dMMR, deficient DNA mismatch repair; MSI-H, microsatellite instability-high; pMMR, proficient mismatch repair; MSS, microsatellite stability. Bold value means that the p value was <0.05.

### Adjuvant Chemotherapy in Deficient Mismatch Repair/Microsatellite Instability-High Gastric Cancer

**Figure 3:**
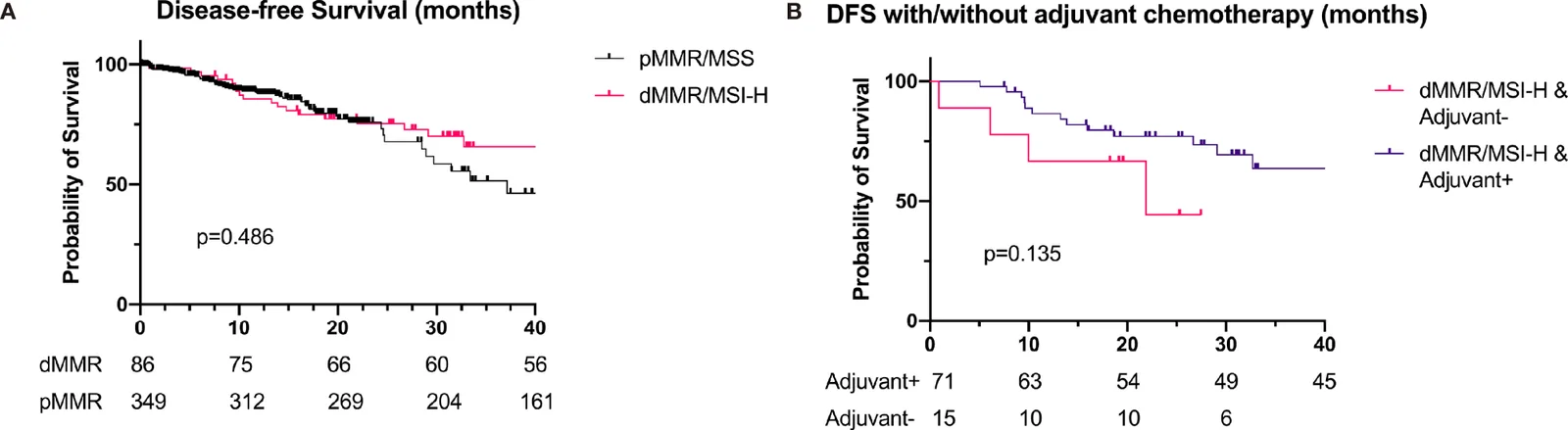
Survival of patients with dMMR/MSI-H (n = 86) and pMMR (n = 349) at stage II and III. **(A)** DFS of patients with dMMR/MSI-H and pMMR (46.9 *vs*. 37.1 months, *p* = 0.486). **(B)** DFS of dMMR/MSI-H patients with/without adjuvant chemotherapy (46.9 *vs*. 21.9 months, *p* = 0.135). dMMR, deficient DNA mismatch repair; MSI-H, microsatellite instability-high; pMMR, proficient mismatch repair; DFS, disease-free survival.

One hundred nineteen (60.7%) dMMR/MSI-H cases were diagnosed at stage II or III, and 117 of them received radical surgery. Eighty-six patients with detail record of adjuvant therapy and follow-up were enrolled for analysis. Seventy-one of them received adjuvant chemotherapy after surgery, and the remaining 15 only received surgery. Compared with the DFS of pMMR GC, the DFS of dMMR/MSI-H was longer (46.9 *vs*. 37.1 months), though the statistical significance was still not reached (*p* = 0.486, **Figure 3**). The disease-free survival (DFS) was 46.9 and 21.9 months for patients with and without adjuvant chemotherapy, respectively, but the difference was not significant (*p* = 0.135, **Figure 3** and **Table 4**).

**Table 4:** Univariable and multivariable Cox proportional hazard models for disease-free survival.

Variables	Categories	Univariate analysis		*p*	Multivariate analysis		*p*
		HR	95% CI		HR	95% CI	
Age				0.293			
	<60	1					
	≥60	0.792	(0.513, 1.223)				
Gender				0.393			
	Male	1					
	Female	1.226	(0.768, 1.958)				
Tumor Location							
	Proximal or EGJ	1			1		
	Middle	0.807	(0.400, 1.628)	0.550	0.936	(0.399, 2.195)	0.878
	Distal	0.958	(0.548, 1.674)	0.879	1.143	(0.573, 2.279)	0.704
	Whole stomach	6.673	(1.527, 28.299)	**0.011**	7.487	(0.810, 69.196)	0.076
	Others	0.655	(0.181, 2.374)	0.520	1.318	(0.286, 6.071)	0.723
TNM stage							
	I	1			1		
	II	5.291	(1.832, 15.279)	**0.002**	4.867	(0.265, 89.298)	0.286
	III	7.415	(2.659, 20.681)	**<0.001**	8.152	(0.285, 233.035)	0.220
	IV	30.245	(5.430, 168.455)	**<0.001**	36.984	(0.285, 2101.473)	0.080
Pathology							
	Adenocarcinoma	1			1		
	Signet ring	1.296	(0.315, 5.330)	0.719	1.145	(0.251, 5.234)	0.861
	Mix (adeno+signet)	1.805	(1.027, 3.174)	**0.040**	1.061	(0.493, 2.285)	0.879
	Others	1.795	(0.650, 4.959)	0.259	2.755	(0.345, 22.025)	0.339
Differentiation				**0.007**			0.145
	High/moderate	1			1		
	Low/undifferentiated	1.848	(1.180, 2.895)		1.685	(0.836, 3.394)	
Lauren classification							
	Diffuse	1			1		
	Intestinal	0.465	(0.259, 0.836)	**0.010**	0.925	(0.384, 2.228)	0.863
	Mix	0.764	(0.432, 1.352)	0.355	0.837	(0.421, 1.663)	0.611
MMR/MS status				0.361			
	dMMR/MSI-H	1					
	pMMR/MSS	1.269	(0.761, 2.114)				
HER2 status				0.451			
	Negative	1					
	Positive	0.672	(0.239, 1.887)				
EBERs				0.711			
	Negative	1					
	Positive	0.821	(0.289, 2.333)				
Adjuvant therapy				0.545			
	No	1					
	Yes	1.166	(0.709, 1.916)				
T stage							
	T1	1			1		
	T2	2.523	(0.600, 10.609)	0.207	1.630	(0.097, 27.410)	0.734
	T3	7.554	(2.338, 24.411)	**0.001**	3.015	(0.171, 53.057)	0.451
	T4	7.256	(2.201, 23.919)	**0.001**	1.892	(0.095, 37.783)	0.676
N stage							
	N0	1			1		
	N1	3.374	(1.738, 6.553)	**<0.001**	1.498	(0.619, 3.626)	0.370
	N2	2.075	(1.012, 4.254)	**0.046**	0.515	(0.136, 1.954)	0.329
	N3	3.227	(1.726, 6.033)	**<0.001**	1.083	(0.304, 3.865)	0.902
Nerves/vessels invasion				**0.013**			0.849
	No	1			1		
	Yes	2.329	(1.191, 4.551)		1.096	(0.425, 2.827)	

HR, hazard ratio; EGJ, esophagogastric junction; MMR, mismatch repair; dMMR, deficient DNA mismatch repair; MSI-H, microsatellite instability-high; pMMR, proficient mismatch repair; MSS, microsatellite stability. Bold value means that the p value was <0.05.

### Chemotherapy and Immunotherapy in Advanced Deficient Mismatch Repair/Microsatellite Instability-High Gastric Cancer

**Figure 4:**
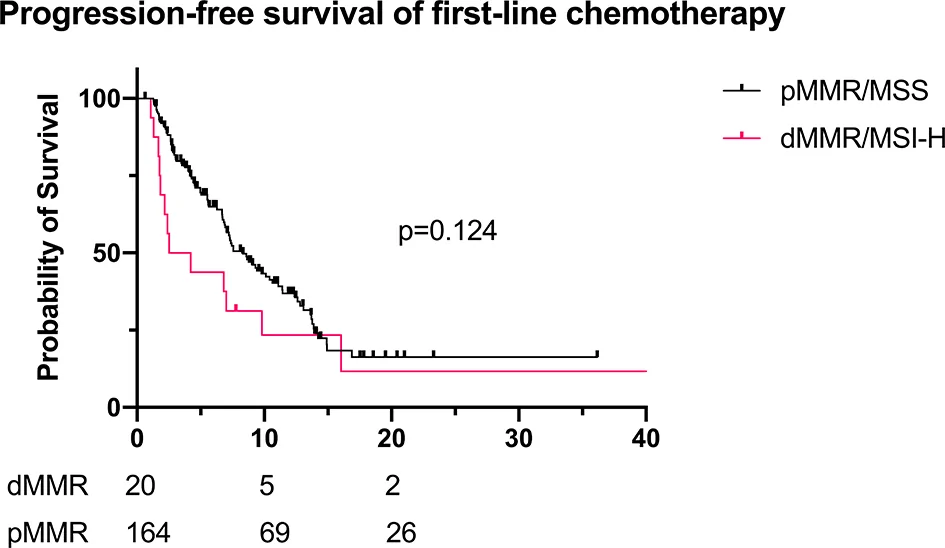
PFS of first-line chemotherapy for recurrent/advanced GC patients (dMMR/MSI-H: n = 20; pMMR: n = 164; mPFS 3.4 *vs*. 8.3 months, *p* = 0.124). PFS, progression-free survival; GC, gastric cancer; dMMR, deficient DNA mismatch repair; MSI-H, microsatellite instability-high; pMMR, proficient mismatch repair; mPFS, median PFS.

The response and progression-free survival (PFS) of first-line chemotherapy (without combination of immunotherapy) are shown in **Table 5**. The objective response rate (ORR) and PFS of dMMR/MSI-H patients were worse than those of pMMR patients (ORR 17.4% *vs*. 26.2%, *p* = 0.361; PFS 3.4 *vs*. 8.3 months, *p* = 0.124, **Figure 4**), though they were not statistically significant. Besides, the disease control rate (DCR) of dMMR/MSI-H patients was remarkably lower than that of pMMR patients (69.6% *vs*. 87.8%, *p* = 0.02).

**Table 5:** The response and progression-free survival of first-line chemotherapy and immunotherapy for dMMR/MSI-H patients.

		mPFS (m)	*p*	ORR (%)	*p*	DCR (%)	*p*
First-line chemotherapy	dMMR/MSI-H	3.4	0.124	17.4	0.361	69.6	0.020
	pMMR/MSS	8.3		26.2		87.8	
Immunotherapy	dMMR/MSI-H	10.6	0.100	57.9	0.016	89.5	0.285
	pMMR/MSS	4.1		25		77.8	

dMMR, deficient DNA mismatch repair; MSI-H, microsatellite instability-high; mPFS, median progression-free survival; ORR, objective response rate; DCR, disease control rate; pMMR, proficient mismatch repair; MSS, microsatellite stability.

**Figure 5:**
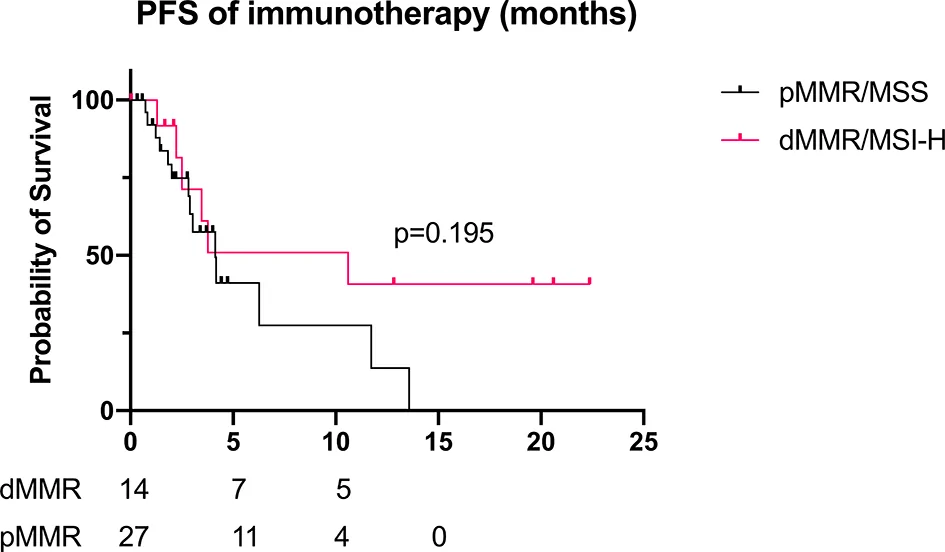
PFS of immunotherapy for recurrent/advanced GC patients (dMMR/MSI-H: n = 14; pMMR: n = 27; mPFS 10.6 *vs*. 4.1 months, *p* = 0.195). PFS, progression-free survival; GC, gastric cancer; dMMR, deficient DNA mismatch repair; MSI-H, microsatellite instability-high; pMMR, proficient mismatch repair; mPFS, median PFS.

Some patients received immunotherapy during their treatment (23 dMMR/MSI-H and 45 pMMR cases). The basic characteristics are shown in **Supplementary Table 1**. For dMMR/MSI-H patients, the ORR of immunotherapy alone and combined therapy was 25.0% and 61.5%, respectively. Since the sample size was too small, the result was not statistically significant (*p* = 0.294). Compared with the pMMR patients, the overall ORR of dMMR/MSI-H patients with immunotherapy was higher (57.9% *vs*. 25.0%, *p* = 0.016). For patients receiving monotherapy, the ORR was 25% for dMMR and 0 for pMMR (*p* = 0.40). For patients receiving combined therapy (immunotherapy + chemotherapy), the ORR was 61.5% and 42.8%, respectively (*p* = 0.092). However, the PFS and DCR of dMMR/MSI-H patients did not differ compared with those of the pMMR group (PFS 10.6 *vs*. 4.1 months, *p* = 0.195; DCR 89.5% *vs*. 77.8%, *p* = 0.285; **Table 5**, **Figure 5**).

## Discussion

This study investigated the clinical and pathological features of dMMR/MSI-H GC and the role of the MMR/MSI status as prognostic and predictive biomarkers of GC. To our knowledge, this is the largest cohort of dMMR/MSI-H GC reported. The prevalence of dMMR/MSI-H in our cohort was 6.6% (4.7%–8.3% in three cancer centers), which was similar to the results reported in previous eastern studies (8%–10%) (4–9), but lower than that in western studies (20% and more) (10, 12, 13). The possible explanation is the difference in gastric carcinogenesis background between eastern and western patients. Besides, MMR/MSI status was associated with several clinical and pathological features such as age, sex, primary site, histology, and Lauren classification. As a result, different clinical and pathological characteristics in eastern and western GC could be responsible of the incidence of MMR/MSI reported in previous studies. In our study, dMMR/MSI-H was associated with older age, female patients, distal location, intestinal subtype, and better differentiation.

Like colorectal cancer, dMMR/MSI-H GC was more often seen in early stage. Whether MMR/MSI status was an independent prognostic factor was still controversial. Some researchers reported it was not a prognostic indicator in GC (4, 18), but others demonstrated that dMMR GC exhibited favorable OS (13, 19, 20). Zhang et al. reported that dMMR status was an independent factor for better prognosis (8). Our study did show that GC patients with dMMR/MSI-H subtype had better OS. However, the prognostic impact of MMR/MSI status was lost on multivariate analysis. As we have mentioned above, dMMR/MSI-H subtype came along with those less aggressive clinical and pathological characteristics such as intestinal histotype and better differentiation. Moreover, dMMR/MSI-H was commonly seen in early TNM stage; therefore, the prognostic value of dMMR/MSI-H may be confounded by other clinical factors, especially the TNM stage.

Although the prognostic value of MMR/MSI status was still controversial, accumulating evidences had identified MSI status as a biomarker of prediction of adjuvant chemotherapy. It was hypothesized that the immunostimulatory environment in dMMR/MSI-H tumors itself can act as a positive prognostic factor for patients receiving radical surgery, so they cannot get further benefit from adjuvant chemotherapy (21–23). Recently, an individual patient data meta-analysis from four large randomized clinical trials (MAGIC, CLASSIC, ARTIST, and ITACA-S) including stage II and III resectable GC patients was performed. It showed that MSI-H status was associated with better DFS and 5-year OS. Besides, patients with MSI-H could not benefit from chemotherapy. Moreover, MSI status was prognostic independent of T/N stage in the study, which implied that adjuvant chemotherapy was not necessary for operable stage II/III GC patients with MSI-H status (14). Several retrospective studies supported this conclusion (6, 8). However, Beghelli et al. found that only stage II MSI-H GC was associated with better prognosis (10). The retrospective study conducted by Tsai et al. showed that the benefit of survival from dMMR was only valid at stage III GC irrespective of the use of adjuvant chemotherapy (24). Marrelli et al. reported that the survival benefit from MSI-H status was only seen in non-cardia GC with Lauren intestinal or tubular/poorly differentiated histology (13). Moreover, Vos et al. reported that though patients with MSI-high tumors had worse pathological response to chemotherapy, they had better OS compared with those with MSS GC (25). Our data differed from the studies mentioned above. According to our analysis, dMMR/MSI-H status was not associated with better DFS. Besides, adjuvant chemotherapy did not affect the DFS or OS in dMMR/MSI-H patients. This result is reasonable given that adjuvant chemotherapy is the standard therapy for stage II/III GC patients after radical surgery, and few dMMR/MSI-H patients in our cohort did not receive adjuvant chemotherapy. The application of adjuvant therapy might attenuate the survival benefit from dMMR/MSI subtype. Besides, several dMMR/MSI-H patients missing adjuvant chemotherapy had severe postoperative complications or worse physiological conditions. Hence, it was hard to discriminate the difference of DFS/OS between dMMR/MSI-H patients with and without adjuvant chemotherapy. Since the sample size of dMMR/MSI-H GC in most studies was too small, and sample bias might exist in retrospective studies, it is too early to withdraw adjuvant chemotherapy for dMMR/MSI-H patients. Randomized controlled study is needed to clarify the role of adjuvant therapy in these patients.

As most dMMR/MSI-H GC patients were diagnosed at an early stage, few studies investigated the role of dMMR/MSI-H status in predicting the efficacy of chemotherapy in recurrent or advanced GC. An et al. retrospectively explored the relation of MMR/MSI status and recurrent GC (5). Although neither MMR/MSI status nor adjuvant chemotherapy was associated with survival after recurrence, dMMR/MSI-H patients who did not receive adjuvant chemotherapy had better response to chemotherapy after recurrence (5). Giampieri et al. suggested that both response rate and PFS of first-line platinum-base chemotherapy were observed in dMMR patients (response rate 66% and PFS 11.2 months for dMMR patients, compared with 19% and 5.0 months for pMMR patients, *p* = 0.0004 and <0.0001, respectively) (26). Our findings were partly in contrast to previous observations. The PFS and ORR of dMMR/MSI-H patients in our study were worse than those in pMMR patients, though the statistical significance was not reached. First, the regimens of chemotherapy were various in our study, which might explain the conflicting results. Second, most recurrent GC patients with dMMR/MSI-H in our study received adjuvant chemotherapy before. According to the result of An et al. (5), these patients might have poorer response to first-line chemotherapy, which might pull down the median PFS and ORR.

dMMR/MSI-H status was associated with several characteristics related to immunotherapy. Defects of DNA replication result in expressions of neoantigens, which result in high mutation burden and act as a potential target for immunecells (27). Attraction of immune cells into tumor environment leads to immune stimulation. It has been reported that high density of intratumoral CD8+ and FoxP3+ tumor-infiltrating lymphocytes were associated with good prognosis (22). Hence, it is reasonable to administrate immune checkpoint inhibitors to enhance the effect of immune stimulation in dMMR/MSI-H tumors (15). In KEYNOTE-059 trial, the response rate of pembrolizumab in GC patients with MSI-H was 57.1%, while it was 9.0% in MSS GC (28). Furthermore, in KEYNOTE-061 trial, anti-PD-1 monotherapy showed better response rate than chemotherapy (paclitaxel) alone in MSI-H GC patients (29). Our study also showed that immunotherapy with or without chemotherapy had better response rate in dMMR/MSI-H patients, though the statistical significance was not reached due to the small sample size. This result suggested that dMMR/MSI-H was a reliable biomarker in predicting the effect of immunotherapy.

Considering the low prevalence of dMMR/MSI-H in GC, this cohort, which enrolled 196 dMMR/MSI-H cases, may be the largest one to date. However, our study has several limitations. First of all, it is a retrospective study, and selection bias inevitably exists. For example, most patients after radical surgery also received standard adjuvant chemotherapy, so it is hard to evaluate the prognostic and predictive roles of adjuvant chemotherapy. Second, the retrospective design and various regimens of chemotherapy used in adjuvant or first-line made it difficult to conduct more subgroup analysis for the effect of chemotherapy or immunotherapy. Third, some important biomarkers associated with immunotherapy, such as tumor mutation burden (TMB) and PD-L1 expression, were not available in this study.

## Conclusion

In summary, dMMR/MSI-H GC patients have specific clinical and pathological characteristics, such as older age, female predominance, distal location in the stomach, earlier TNM stage, intestinal subtype, better differentiation, and more negative HER2 status. Although dMMR/MSI-H is a predictive factor of immunotherapy in advanced stage, it was not an independent prognostic factor in GC. Moreover, the predictive and prognostic value of chemotherapy for dMMR/MSI-H GC in adjuvant or first-line setting is not clear, which should be further investigated in prospective clinical trials.

## Data Availability Statement

The datasets presented in this study can be found in online repositories. The names of the repository/repositories and accession number(s) can be found in the article/**Supplementary Material**.

## Ethics Statement

The studies involving human participants were reviewed and approved by the ethics committee of Sun Yat-sen University Cancer Center (B2020-335-01). Written informed consent for participation was not required for this study in accordance with the national legislation and the institutional requirements.

## Author Contributions

M-ZQ and F-HW designed the study. W-LG, YM, and Y-HC made the data analysis. J-YX, J-YL, L-QY, and YTS collected the data. Z-WZ, T-SL, Y-QZ, and R-HX provided the concept. W-LG and M-ZQ wrote the manuscript. All authors contributed to the article and approved the submitted version.

## Funding

This work was supported by the National Natural Science Foundation of China (grant numbers 82073377 and 81772587), Natural Science Foundation of Guangdong (2021A1515012439), Guangdong Esophageal Cancer Institute Science and Technology Program (grant number M201809), CSCO-HengRui Oncology Research Fund (grant number Y-HR2018-184), and the third outstanding young talents training plan and Medical Scientist program of Sun Yat-sen University cancer center.

## Conflict of Interest

The authors declare that the research was conducted in the absence of any commercial or financial relationships that could be construed as a potential conflict of interest.

## Publisher’s Note

All claims expressed in this article are solely those of the authors and do not necessarily represent those of their affiliated organizations, or those of the publisher, the editors and the reviewers. Any product that may be evaluated in this article, or claim that may be made by its manufacturer, is not guaranteed or endorsed by the publisher.
