# Supplementary material for: The Impact of Mismatch Repair Status on Prognosis of Patients With Gastric Cancer: A Multicenter Analysis
Source: Front Oncol. 2021 Nov 25;11:712760. doi: 10.3389/fonc.2021.712760 (PMC8655239; doi:10.3389/fonc.2021.712760)
Supplement: Supplementary file 2 [file Table_1.docx]

Supplementary Table 1. Basic characteristics of patients who received immunotherapy during their treatment.

| Variables | N(%) | | p |
| --- | --- | --- | --- |
|  | dMMR | pMMR |  |
| Gender |  |  | 0.442 |
| Male | 10 (43.5) | 24(53.3) |  |
| Female | 13 (56.5) | 21(46.7) |  |
| Age |  |  | 0.412 |
| <60 | 13 (56.5) | 30(66.7) |  |
| ≥60 | 10 (43.5) | 15(33.3) |  |
| Pathology |  |  | 0.741 |
| Adenocarcinoma | 17 (73.9) | 36 (80.0) |  |
| Signet ring | 1 (4.3) | 2 (4.4) |  |
| Mix(adeno+signet) | 1 (4.3) | 5 (11.1) |  |
| Others | 2 (8.7) | 2 (4.4) |  |
| NA | 2 (8.7) | 0 (0) |  |
| Differentiation |  |  | 0.83 |
| High/Moderate | 7 (30.4) | 18 (40.0) |  |
| Low/Undifferentiated | 11 (47.8) | 25 (55.6) |  |
| NA | 5 (21.7) | 2 (4.4) |  |
| Lauren classification |  |  | 0.249 |
| Diffuse | 5 (21.7) | 16 (35.6) |  |
| Intestinal | 2 (8.7) | 11 (24.4) |  |
| Mix | 6 (26.1) | 8 (17.8) |  |
| NA | 10 (43.5) | 10 (22.2) |  |
| HER2 status |  |  | 0.164 |
| Negative | 20 (87.0) | 36 (80.0) |  |
| Positive | 0 (0) | 6 (13.3) |  |
| NA | 3 (13.0) | 3 (6.7) |  |
| Treatment |  |  | 0.299 |
| 1st line | 13 (56.5) | 20 (44.4) |  |
| 2nd line | 4 (17.4) | 10 (22.2) |  |
| 3rd line | 2 (8.7) | 8 (17.8) |  |
| 4th or 5th line | 3 (13.0) | 1 (2.2) |  |
| NA | 1 (4.3) | 6 (13.3) |  |
| Combination |  |  | 0.267 |
| Immunotherapy alone | 8 (34.8) | 10 (22.2) |  |
| Immunotherapy+ Chemo | 15 (65.2) | 35 (77.8) |  |
